# Radical chest wall resection and modified reconstruction technique for solitary internal mammary lymph node recurrence in breast cancer

**DOI:** 10.1016/j.ijscr.2019.03.021

**Published:** 2019-04-06

**Authors:** Ikram Ulhaq Chaudhry, Thabet Algazal, Zahra Alhajji, Ahsan Cheema, Hadi Al Mutairi, Fasisal Azem

**Affiliations:** Department of Thoracic Surgery and Oncology, King Fahad Specialist Hospital Dammam, Saudi Arabia

**Keywords:** Breast cancer, Surgery, Radiotherapy, Metastasis, Internal mammary lymph node, Reconstruction

## Abstract

**Introduction:**

Although the incidence of internal mammary lymph node recurrence rate in breast cancer is low but still it is the second most common drainage site after axilla. Patients with solitary internal mammary lymph node (IMLN) recurrence have overall better prognosis. The role of chemo and radiotherapy in internal mammary lymph node involvement with breast cancer is still controversial. Radical surgical resection and reconstruction remains mainstay for good prognosis.

**Presentation of case:**

Here in we present a case of a 32 year old female with breast cancer who had left mastectomy followed by adjuvant chemo radiotherapy treatment for adenocarcinoma of breast in 2008. She presented with upper left parasternal pain in 2009. Computed tomographic scan (CT) of her thorax showed internal mammary lymph node enlargement, likely metastasis. We performed modified surgical reconstruction after enbloc radical resection of part of manubrium; hemi sternum, chest wall and left parasternal IMLN. Patient remained disease free to date.

**Discussion:**

There is no standard treatment after IMLN metastasis. Previous surgical studies reported no survival benefit with ERM, but is there any role of adjuvant locoregional radiotherapy or systemic therapy to prevent relapses in IMLN is a matter of debate.

**Conclusion:**

To the best of our knowledge this is first case ever managed with radical enbloc surgical resection and modified reconstruction of chest wall using such technique. Our reconstruction technique provides better chest wall stability with minimal risk of plate dislodgement or excursion and at the same time provides good cosmoses and better survival.

## Highlights

• Solitary internal mammary lymph node metastasis in breast cancer patient.
• Radical enbloc surgical resection of part of manubrium, hemi sternum two ribs and internal mammary lymph node.
• Modified chest wall reconstruction technique provides better chest wall stability and cosmoses.
• There is minimal chance of MMS plate excursion or dislodgment which is the most dreadful complication in chest wall reconstruction.
• To date the patient is disease free.

## 1 Introduction

The extended radical mastectomy (ERM), which includes removal of internal mammary lymph node, was considered standard operation until 1970. This practice was abandoned as many studies concluded that prophylactic removal of IMLN had shown no prognostic impact. Internal mammary lymph node involvement is seen only in 8–27% and 4–65% during sentinel lymph node biopsy and during surgery respectively [[1], [2], [3]]. Surgical practice for breast cancer has been changed with advances in receptor based endocrine therapy and chemotherapy. Modern trend is more towards breast conserving surgery. This work has been reported in line with the SCARE criteria [4].

## 2 Presentation of case

**Fig. 1:**
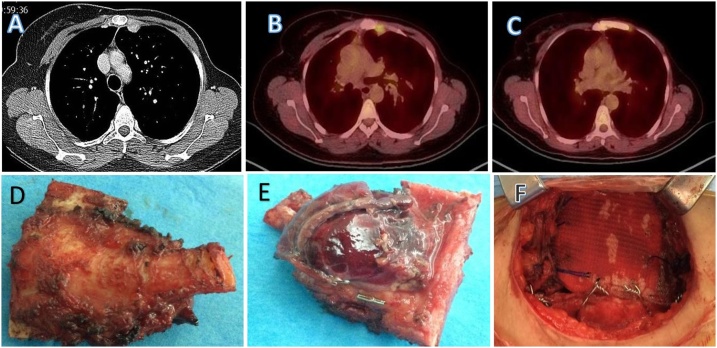
A: CT scan of Thorax showing internal mammary lymph node metastasis. B Preoperative PET scan showing FDG avid internal mammary lymph node C Post operative PET scan showing no recurrence. D Resected specimen ventral view of enbloc resection of mammary node with bony structures E Dorsal view of the resected specimen. F Final reconstruction.

A 32 years old female underwent left mastectomy for invasive ductal carcinoma of the breast in 2008 followed by adjuvant chemotherapy using 8 cycles of cyclophosphamide, epirubicin, 5-fluorouricil and docetaxel. In 2009 she presented with left parasternal pain. CT scan of thorax and Positron emission tomography (PET) scan, revealed a solitary left IMLN enlargement measuring 2.5 cm (Fig. 1A & B). A CT guided biopsy confirmed, “Metastatic Adenocarcinoma of breast origin”. Multidisciplinary team including oncologist, radiologist, pathologist, breast surgeon and thoracic surgeon recommended for surgical resection. Radical enbloc surgical resection of solitary internal mammary metastatic mass along with adjacent bony structures was carried out and chest wall defect was reconstructed by our modified reconstruction technique with excellent prognosis.

**Fig. 2:**
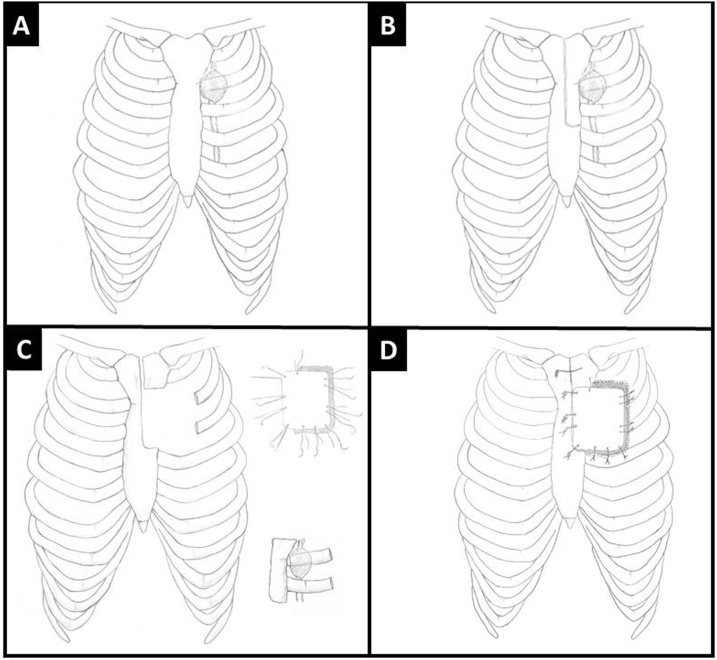
(A) Drawing showing internal mammary lymph node (B) showing the residual defect after enblock resection of part of manubrium, hemi sternum ribs and IMLN (C&D) showing our modified reconstruction technique.

Partial median sternotomy was performed. Skin and muscle flaps were raised on left side, manubrium and upper sternum (3 cm) was divided. Internal mammary vascular pedicle was ligated. Part of manubrium and sternum was resected enbloc with medial part of second and third rib (Fig. 1D & E). Chest wall defect was measured, methyl methacrylate (MMS) marlex mesh plate was prepared and molded according to the shape of defect. Holes were drilled in the manubrium, sternum, 3 & 4 rib and corresponding holes were also drilled in the MMS plate. MMS plate was then anchored by 5 mm wires to sternum and manubrium and remaining 3rd and 4th rib while mesh was sutured to the surrounding tissues with a 2-0 prolene suture. The medial side of the plate was anchored to the manubriosternum with wires had no mesh (Fig. 1F). The chest wound was closed over by approximating the soft tissues and pectorals major muscles. Skin was closed without the use of myocutaneous flap. Recent PET scan showed no recurrence (Fig. 1C). All surgical procedure steps are shown in illustrations drawings in Fig. 2A–D.

## 3 Discussion

IMLN metastases are more common with inner and central quadrant breast tumors as compared to the lateral quadrant. Extended radical mastectomy using Halsted classical radical mastectomy procedure that included excision of pleura and IMLN was abandoned in the 1970s. For staging and therapeutic purposes a low rates of IMLN metastases in the absence of concomitant axillary lymph node (ALN) metastases were found. This therapeutic procedure was abandoned because removal of all IMLN fails to show any improvement in the prognosis. There were no benefits in survival or local control with extended radical mastectomy (EM) as compared to radical or modified radical mastectomy. But more surgical trauma and pleura-related complications were reported [5,6]. The surgical trends have since moved towards breast conserving surgery as that can prevent an immense emotional and psychological trauma to patients and family. Breast-conserving surgery with sentinel node biopsy is now a widely accepted procedure for early breast cancer. It is well known that lymphatic spread from the breast cancer occurs in axillary, supraclavicular and IMLN [[7], [8], [9], [10], [11]]. There is a debate about the value of harvesting extra- axillary sentinel node shown on the lymphoscintigram. Surgical resection of IMLN or radiation to internal mammary chain has not shown any survival beneﬁt at the time of breast surgery. Therefore, involved IMLN are often not detected and left untreated. Diagnosis based on clinical symptoms after recurrence in the IMLN is seen only in 0.1% of breast cancer patients [12,13]. There is no standard treatment after IMLN relapse. There is a greater interest in preventing the relapse and so of adjuvant treatments. Previous surgical studies reported no survival benefit with ERM, but are there any role of adjuvant locoregional radiotherapy or systemic therapy to prevent relapses in IMLN is a matter of debate.

Early Breast Cancer Trialists' Collaborative Group (EBCTCG) had answered this by reporting the importance of loco regional control and its impact on long term survival [14]. This benefit of loco regional control improving the long-term survival was limited to trials that used systemic therapies, which was not a routine in the earlier surgical studies. So, the survival contribution from treating IMLN is still not clear. Systemic adjuvant chemotherapy is well known to improve survival and is traditionally offered to selected patients with localized breast cancer after assessing the prognostic clinical risk factors. On the other hand, patients with hormone receptor-positive tumors are nearly always considered for adjuvant endocrine therapy lasting for 5–10 years. Radiation of the chest wall and regional lymph nodes following mastectomy is recommended in selected high-risk patients but the value of IMLN irradiation in patients with positive IMLN remains unclear as the prognostic significance of IMLN recurrences is scarce and controversial. Radiation in this area also increases the risk of cardiac morbidity. Patients who relapse with IMLN metastases, usually have distant metastases but if the imaging scans does not show distant metastases, then loco-regional treatment of the isolated IMLN recurrence, by surgery or radiotherapy, could be indicated. Most of those patients would have adjuvant radiation therapy after breast conserving surgery. Therefore, surgery will be the treatment of choice for isolated IMLN recurrences. Previous studies have reported the chest wall resection as safe and effective surgical procedure, when performed by specialized surgeons. Albertus N et al reported from their retrospective study of 29 patients, who had isolated IMLN metastases after initial primary treatment for breast cancer. Median duration of isolated IMLN relapse was 5.1 years after treatment for a primary breast cancer. The median follow- up after chest wall resection was 18.4 months. The 3-year overall and disease-free survival was 59.2% and 8.6%. The median survival was 40.7 months. Systemic therapy before chest wall resection was the most signiﬁcant factor for survival and radicality of the resection was a significant factor for local recurrence-free survival and disease-free survival [15]. In medical literature reported overall 5 years survival rate is 29–56%, after wide local excision, chest wall resection, sternal resection, and chest wall reconstruction [[16], [17], [18], [19], [20], [21]]. Further studies showed the overall 5 year survival is 29–74% and five year disease free survival is 13–67% (median 11–39 months) [22,23]. Chest wall resection is a safe and effective treatment for isolated breast cancer recurrences in the IMLN chain. Surgically treated patients have a fair survival and some of them are even cured. As these patients can have a long term survival, so the surgical reconstruction techniques are extremely important for patient self-esteem and cosmoses.

## 4 Conclusion

In conclusion we present a 32 years female patient with solitary IMLN metastases treated with radical enblock surgical resection and modified reconstruction of chest wall. To the best of our knowledge this is first case ever managed with such technique of chest wall reconstruction. Our reconstruction technique provides better chest wall stability with minimal risk of plate dislodgement or excursion and at the same time provides good cosmoses and better survival.

## Conflicts of interest

There is a no conflict of interest in this paper.

## Sources of funding

There is no funding/grant involved in this case report.

## Ethical approval

Institutional review board (IRB) approval achieved. Ref 018-011 Dated 5/12/18.

## Consent

A copy of the written consent is available for review by the Editor in Chief of the journal upon request.

## Author’s contributions

Ikram Ulhaq Chaudhry: Operating surgeon drafting the article, Critical revision and final approval of the article.

Thabet Al gazal MD: Pictures and imaging.

Zahra Alhajji: Draw illustrations.

Ahsan Cheema, MD: Conception and design, References.

Hadi Al Mutairi: Assisted surgery.

Faisal Azam MD: Medical oncologist who gave chemotherapy and gave oncological opinion in manuscript.

## Registration of research studies

No clinical trial or observational research is involved. It is a surgical technique.

## Guarantor

The corresponding author Dr Ikram Chaudhry is the guarantor.

## Provenance and peer review

Not commissioned, externally peer-reviewed.
